# The relationship between locus of control and depression: A cross-sectional survey with university students in Botswana

**DOI:** 10.4102/sajpsychiatry.v25i0.1221

**Published:** 2019-02-19

**Authors:** Tsholofelo Khumalo, Ilse E. Plattner

**Affiliations:** 1 Department of Psychology, University of Botswana, Botswana

## Abstract

**Background:**

Research has consistently revealed a positive association between external locus of control and depression. Little, if any, research has investigated locus of control and depression in the sociocultural context of Botswana.

**Aim:**

To explore the relationship between locus of control and depression among undergraduate students in Botswana and to determine the impact of age and gender on this relationship.

**Setting:**

University of Botswana.

**Methods:**

A sample of 272 students was surveyed through a self-administered questionnaire, which included the Levenson’s multidimensional locus of control scale, the Beck Depression Inventory-II and demographic questions. Data analysis utilised descriptive statistics, correlation analysis, independent samples *t*-tests and standard multiple regression analysis.

**Results:**

Of the 272 participants, 47.3% scored low (minimal) levels of depression, 23.4% scored mild levels, 18.0% scored moderate levels and 11.3% scored severe levels of depression. Students who believed that they were in control of events in their lives were less likely to present with depressive symptoms (*r* = -0.29, *p* = 0.000), while students who believed that chance (*r* = 0.45, *p* = 0.000) or powerful others (*r* = 0.40, *p* = 0.000) controlled their lives were more likely to have high depression scores. Both internal and external locus of control, together with age, explained 31% of the variance in depression scores; gender made no significant contribution to levels of depression.

**Conclusion:**

The study results draw attention to locus of control as one of the cognitive variables associated with depression. Further research is needed to determine how locus of control can be addressed in the treatment and prevention of depression in university contexts.

## Introduction

Mental health problems among university students have been widely studied.^1^ University life can be straining for students owing to potential stressors such as having to adjust to a new environment, being away from the familiarity and support of the parental home, having to live on a tight budget, having to complete assignments within limited time frames and/or having to cope with fluctuations in romantic relationships.^2^ While for some students the challenges of university life might be an opportunity for personal growth, for others these challenges may prompt mental health problems such as depression.^3,4^ Whether or not university life contributes to students developing depression will depend on various genetic, neurobiological and psycho-social factors,^5,6^ but may also be influenced by students’ beliefs that they have little or no control over events occurring in their lives. Such beliefs are referred to as ‘locus of control’.

The construct of locus of control was introduced by Julian Rotter as part of his social learning theory.^7^ Locus of control refers to individuals’ generalised expectancy or belief as to whether events in their lives are controlled by their behaviour and abilities (internal locus of control) or by external forces such as powerful others, chance, fate or luck (external locus of control). Although the extent to which people expect events to be controlled by themselves or by external factors may vary between situations and events, people tend to display a more generalised locus of control when interpreting events in their life.^7^

Some studies have established a relationship between locus of control and depression.^8,9^ Peterson^10^ predicted that people with internal locus of control would be more likely to present with higher levels of depression as depressed people tend to blame themselves for failure.^11^ Contrary to such prediction, research consistently has shown that external locus of control (and not internal locus of control) was positively associated with depression.^12,13,14^ Associations between locus of control and depression were also established in student samples from various parts of the world. For example, an American study found that college students who displayed internal locus of control had lower depression scores than students with external locus of control.^15^ In a more recent study with American undergraduate students, external locus of control predicted the variance in depression scores while internal locus of control did not.^16^ A study with female college students in India found that depression was negatively associated with internal locus of control, whereas it was positively associated with students’ beliefs that powerful others and chance would control events.^17^ Similarly, a study with undergraduate students in Jordan established a negative relationship between depression and internal locus of control, whereas the belief that events would be controlled by chance was positively associated with depression.^4^ A South African study with local and international graduate students found that high levels of external locus of control were associated with low levels of general well-being.^18^ While it may be assumed that cultural differences would moderate the effect of locus of control on depression, a meta-analysis of various locus of control research established that studies conducted in collectivist societies revealed as much a positive association between external locus of control and depression as did studies conducted in the so-called individualistic societies.^14^

The theory of locus of control helps to explain, at least to some extent, why some students are able to successfully adapt to the demands of university life while others become vulnerable to depression. When faced with challenges, students who believe to a large extent that they are in control of events in their lives will feel encouraged to become proactive. Students who believe that they have little control and that the control lies with powerful others or chance are likely to experience emotional distress and become passive in their behaviour. It appears that the belief that one has little or no control over events that happen in one’s life affects a person in a similar way as actual lack of control does, that is, it causes feelings of helplessness, passivity, loss of interest and feelings of hopelessness,^19^ all of which could make a person vulnerable to developing depression.

The literature search did not reveal any research that had investigated the relationship between locus of control and depression in the sociocultural context of Botswana. While no official statistics about the prevalence and distribution of depression are available for Botswana, previous studies identified relatively high prevalence of depression among university students.^3,20^ The study aimed to explore the association between locus of control and depression in a sample of undergraduate students in Botswana. The study followed Levenson’s^21^ proposition to consider internal and external locus of control as two separate variables and to pay attention to the extent to which people believe that events in their lives are controlled by themselves or external forces. With regard to the latter, Levenson^21^ further proposed to differentiate between beliefs in powerful others and beliefs in chance. It was hypothesised that higher levels of internal locus of control would be associated with lower levels of depression and that higher levels of external locus of control referring to both powerful others and chance would be associated with higher levels of depression. Considering that the literature reports that women are more prone to depression than men^22^ and that in some developing countries younger people are less depressed than older people,^22^ the study also hypothesised that locus of control together with gender and age would predict levels of depression. The study contributes towards knowledge about the role of cognitive factors in the development of depressive symptoms, with particular reference to the social ecology of Botswana.

## Method

### Study design

A cross-sectional survey was conducted within the quantitative research paradigm.

### Participants and procedure

Applying convenience sampling method, the researchers distributed a self-administered questionnaire in classes attended by undergraduate students from various disciplines within the University of Botswana. The participants completed the questionnaires individually in the lecture venues and returned them to the researchers in the class. In total, 335 questionnaires were distributed, of which 303 questionnaires were returned. Twenty-four questionnaires were excluded from data analysis because they were incomplete over large parts; seven questionnaires were not considered because the respondents were below the age of 18 years and no informed consent was obtained from their parents. Thus, the final sample comprised 272 participants.

### Instruments

The self-administered questionnaire contained two scales assessing locus of control and depression, respectively, and questions to determine participants’ gender and age. In order to describe characteristics of the sample, additional questions were asked about participants’ year of study, faculty enrolement and parents’ level of education as an indicator of participants’ socio-economic background.

Locus of control was assessed using Levenson’s multidimensional locus of control scale.^23^ This scale was chosen because it measures locus of control as a multidimensional construct unlike the Rotter scale that measures locus of control as a one-dimensional construct. The Levenson scale measures the extent to which people believe that events in their lives are controlled by themselves (internal locus of control), or by chance and by powerful others (external locus of control). This scale contains 24 items that are divided into three subscales (eight items each), one measuring internal locus of control, one measuring the ‘chance’ dimension of external locus of control and the third one measuring the ‘powerful others’ dimension of external locus of control. Each item has six response categories and is scored on a scale ranging from ‘strongly disagree’ (scored as −3) to ‘strongly agree’ (scored as +3). To avoid negative total scores, 24 scores per subscale are added to the scores obtained from the responses; total scores can range from 0 to 48 scores per subscale. Levenson reported good convergent validities for the scale and reliability values ranging between mid 0.60s and mid 0.70s.^23^

Depression was measured with the Beck Depression Inventory-II (BDI-II).^24^ This inventory is suitable for use in a non-psychiatric population including university students^25^ and screens for symptoms of depression during a period of 2 weeks prior to and including the day of administration. The BDI-II contains 21 symptoms (e.g. sadness) and each of them is presented with four statements scored on a scale from 0 (e.g. I do not feel sad) to 3 (e.g. I am so sad or unhappy that I can’t stand it). Total scores can range from 0 to 63, indicating the severity of the depression. Scores ranging from 0 to 13 indicate minimal depression, 14 to 19 mild depression, 20 to 28 moderate depression and 29 to 63 severe depression.^24^ High convergent and discriminant validities and a strong reliability of alpha = 0.93 for non-clinical samples have been reported for the BDI-II.^24^ The BDI-II has been employed before in the Southern African context^26^ as well as in the Botswana context^3,20^ and was, therefore, regarded as appropriate for the present study.

### Data analysis

Data were analysed utilising descriptive statistics, independent samples *t*-tests (to determine gender differences), Pearson’s product–moment correlation coefficient (to determine associations between locus of control, age and depression) and standard multiple regression analysis (to determine whether internal and external locus of control [powerful others, chance], gender and age predicted depression).

## Ethical consideration

Participation in the study was anonymous and voluntary and participants were assured that they could withdraw from filling out the questionnaire at any time. Ethical clearance was obtained from the Department of Psychology Ethics Board for Student Research at the University of Botswana. Considering possible distressing effects of filling out the BDI on the students, the researchers debriefed all participants and offered them information about where they could seek psychological help if they felt that the questionnaire had evoked some emotional distress. Although the researchers had announced in classes that students under the age of 18 years should not participate in the study, seven under-aged students filled out the questionnaire. Owing to the anonymity of the participants, the researchers were not able to identify these seven students in order to provide them with additional assistance. However, the seven participants were part of the debriefing about where to obtain psychological help.

## Results

The study included a total of 272 participants. Their average age was 20.10 years (mean; standard deviation [s.d.] = 2.84) and most of them were female students (80.2%). Participants were first-year (42.5%), second-year (30.8%), third-year (12.8%) and fourth-year (13.9%) undergraduate students enrolled in the Faculties of Social Sciences (69.9%), Business (23.8%), Humanities (2.2%), Education (2.2%), Health Sciences (1.5%) and Science (0.4%). Almost half of the participants’ mothers or female guardians (45.7%) had completed tertiary level education; 44.9% reported the same for their fathers or male guardians.

In the study, internal consistency reliability was low for the internal locus of control subscale (α = 0.44) but acceptable for the subscales measuring the ‘chance’ dimension (α = 0.69) and the ‘powerful others’ dimension of external locus of control (α = 0.70). A Cronbach’s alpha of 0.90 was obtained for the BDI-II, suggesting a strong internal consistency reliability.

Table 1 presents average locus of control and depression scores by gender and age. A mean score of 34.85 (s.d. = 6.69) was obtained for internal locus of control. For external locus of control, the mean scores were 18.33 (s.d. = 9.77) for locus of control (chance) and 20.23 (s.d. = 8.93) for locus of control (powerful others).

**TABLE 1:** Average locus of control and depression scores by gender and age.

Variable	Mean	s.d.	Gender		Age	
			*t*	*p*	*r*	*p*
Internal locus of control	34.85	6.69	0.430	0.667	−0.04	0.549
External locus of control (chance)	18.33	9.77	1.104	0.273	−0.07	0.265
External locus of control (powerful others)	20.23	8.93	0.138	0.890	0.08	0.220
Depression	15.23	10.42	1.380	0.169	−0.20	0.001

s.d., standard deviation.

The mean score for depression was 15.23 (s.d. = 10.42); the percentage of participants who scored at minimal level of depression was 47.3% and 23.4% at mild depression, 18.0% at moderate depression and 11.3% at severe level of depression.

Gender and age made no difference in participants’ locus of control scores. Gender also made no difference in participant’s depression scores. However, age had a weak but statistically significant negative association with depression. Younger participants were more likely to have higher depression scores (*r* = −0.20, *p* = 0.001; Table 1).

Table 2 presents associations between locus of control and depression. The results show that the correlation between internal locus of control and depression was negative though weak (*r* = -0.29, *p* = 0.000), while both external locus of control (chance) and external locus of control (powerful others) were positively associated with depression; the correlations were moderate (*r* = 0.45, *p* = 0.000; *r* = 0.40, *p* = 0.000).

**TABLE 2:** Associations between locus of control and depression.

Dimensions of locus of control	Depression	
	*R*	*p*
Internal locus of control	−0.29	0.000
External locus of control: chance	0.45	0.000
External locus of control: powerful others	0.40	0.000

To determine whether or not locus of control predicted depression, standard multiple regression analysis was performed. A model was tested that included (1) internal locus of control, (2) external locus of control (chance), (3) external locus of control (powerful others), (4) gender and (5) age as predictor variables (Table 3). This model explained 30.7% of the variances in depression scores (*R*^2^ = 0.307, adjusted *R*^2^ = 0.285, *F*[5, 211] = 18.19, *p* = 0.000). External locus of control (chance) made the largest unique contribution (beta = 0.28). External locus of control (powerful others) (beta = 0.20), internal locus of control (beta = -0.25) and age (beta = -0.17) also made significant contributions, while gender did not significantly contribute to depression.

**TABLE 3:** Locus of control, gender and age as predictors of depression.

Variable	*R*^2^	Adjusted *R*^2^	Beta	*F*	*df*	*p*
Model	0.307	0.285	-	18.19	5.211	0.000
Internal locus of control	-	-	−0.25*	-	-	-
External locus of control (chance)	-	-	0.28*	-	-	-
External locus of control (powerful others)	-	-	0.20*	-	-	-
Gender	-	-	−0.07	-	-	-
Age	-	-	−0.17*	-	-	-

* *p* ≤ 0.05

## Discussion

This study aimed to explore the relationship between locus of control and depression in a sample of university students in Botswana. The results revealed that internal locus of control was negatively associated with depression. The two dimensions of external locus of control (chance and powerful others) were positively associated with depression. The associations between locus of control and depression were moderate and lower for internal locus of control than for the two dimensions of external locus of control.

Both low internal and high external locus of control predicted depression; together with age they explained about 31.0% of the variances in the participants’ depression scores. These results are consistent with findings from various other studies.^12,13,14^

While there are various genetic, neurobiological and psycho-social factors contributing to depression, the results draw attention to locus of control as one of the cognitive variables playing a significant role in depression. The results suggest that when students believe that they are in control over events in their lives they are less likely to present with depressive symptoms. One can assume that such belief encourages students to address stressful events proactively, which prevents them from engaging in negative thoughts about themselves, the world and the future^11^ as well as feeling helpless, and becoming passive and indecisive. For students with high internal locus of control, events and challenges that are typically encountered in university life may become an opportunity for personal growth instead of triggering or activating depression. Many university students are constantly exposed to a variety of academic, financial and relational challenges. The challenges include submitting assignments on time, worrying about passing tests and examinations, the adequacy of a monthly stipend, the ability to afford a desirable lifestyle and coping with break-ups in their romantic relationships.^2^ While not all challenges are within the personal control of students, a student’s perception of whether or not these challenges and stressors are within his or her control is likely to influence whether he or she is likely to proactively approach the challenges or not.

In this study, the belief that ‘chance’ would control events in one’s life was slightly more strongly associated with depression than the belief that ‘powerful others’ would control events in one’s life. This result differs from findings in other studies where the belief in ‘powerful others’ produced slightly stronger associations with depression than the belief in ‘chance’.^12^ Research would have to explore factors contributing to individuals believing in events in their lives being controlled by chance or by powerful others. Research would also have to explore whom the ‘powerful others’ are said to be. It could be that sociocultural differences determine the strength of the association between beliefs in chance or in powerful others and depression. In a study of Jordanian students, Zawawi and Hamaideh,^4^ for example, established a significant association between depression and the belief in chance, but not between depression and the belief in powerful others. According to Levenson:^21^

[*P*]eople who believe that the world is unordered (chance) would behave and think differently than people who believe that the world is ordered but that powerful others are in control. (p. 398)

Beliefs in chance could potentially result in more feelings of helplessness and produce more depressive symptoms as chance is basically not controllable, whereas powerful others, depending on who they are, could, in principle, be influenced. According to Levenson,^21^ the expectation that chance will control events in one’s life also includes the belief in fate. In the Botswana context where, for example, traditional beliefs in mystical powers, witchcraft and evil spirits are apparently quite existent,^27^ such beliefs could nurture an external locus of control and a fatalistic attitude, leading to feelings of helplessness, passivity and giving-up behaviour. More in-depth research is required to explore how sociocultural factors and belief systems in Botswana contribute to some people approaching life predominantly with an internal locus of control while others approach life predominantly with an external locus of control.

In this study, 18.0% of the participants scored a moderate level of depression and 11.3% scored a severe level of depression. These percentages are within the range of depression prevalence found in other studies with university students in the region,^3,28,29^ although the results vary widely. Younger students had higher depression scores than older students, which suggests that for some students the beginning of their student life was already overshadowed by depressive symptoms, which were likely to affect their academic performance^30^ and which could result in them dropping out before completing their studies.

While the prevalence of depression in this sample does not permit us to draw conclusions about its representativeness in the larger student community, it nevertheless calls for psychological and psychiatric interventions. The results also indicate a need for preventative strategies that attend to students with external locus of control before they present with depressive symptoms. Rotter^7^ asserted that locus of control is learnt. As one develops and gains experience, a person learns to differentiate between events that are a consequence of his or her behaviour and events that are controlled by external forces. Student counselling centres could offer prevention programmes that sensitise students and empower them psychologically to modify their locus of control where appropriate. Such programmes should take into account the difference between personal and systemic (structural) circumstances to avoid depressing a student who is facing stressors that are beyond his or her control. It would be important for students to become sensitised to the adequacy of an internal or external locus of control taking into account the situational circumstances. In this way, students would be better equipped to maintain their mental health and to cope proactively with stressful events that characterise university life.

### Limitations

The study had a few limitations. Firstly, the sampling method does not allow for the generalisation of the results to other populations or other university students. Secondly, the reliability of the study could have been impacted negatively by the use of self-report measures. Thirdly, the study did not consider mediating and moderating variables that could have influenced the association between locus of control and depression. More research is necessary to address these limitations.

## Conclusion

This study aimed at exploring the relationship between locus of control and depression among undergraduate students in Botswana. The results draw attention to locus of control as one of the cognitive variables that play a significant role in depression. The results suggest that students who display internal locus of control are less likely to be depressed, whereas students who display external locus of control are more likely to present with depressive symptoms. Depression has negative implications for students’ academic success, future employment, future relationships and their happiness in life. It is worthwhile to consider locus of control as one of the significant variables when addressing depression as a mental health problem among university students. Further research is needed to determine how locus of control can be addressed successfully in the treatment and prevention of depression within university contexts.
